# Quality assurance and improvement in oncology using guideline-derived quality indicators – results of gynaecological cancer centres certified by the German cancer society (DKG)

**DOI:** 10.1007/s00432-022-04060-8

**Published:** 2022-06-03

**Authors:** E. Griesshammer, S. Wesselmann, M. W. Beckmann, C. Dannecker, U. Wagner, N. T. Sibert, R. Armbrust, J. Sehouli

**Affiliations:** 1 Charité-University Hospital Berlin, Berlin, Germany; 2 German Cancer Society e.V., 14057 Berlin, Germany; 3 Department of Obstetrics and Gynecology, University of Erlangen, 91054 Erlangen, Germany; 4 Department of Obstetrics and Gynecology, University Hospital Augsburg, 86156 Augsburg, Germany; 5 Clinic for Gynecology, Gynecological Oncology and Gynecological Endocrinology, University Hospital of Giessen and Marburg (UKGM), Marburg, Germany; 6 Department of Gynecology with Center for Oncological Surgery, Charité-University Hospital Berlin, Berlin, Germany

**Keywords:** Quality indicators, Quality assurance, Health service research, Certification

## Abstract

**Purpose:**

Based on the example of Gynaecological Cancer Centres (GCCs) certified by the German Cancer Society, this study evaluates the results of medical-guideline-derived quality indicators (QIs) for cervical cancer (CC) and ovarian cancer (OC), examines the development of indicator implementation over time as well as the status of guideline-compliant care and identifies improvement measures.

**Methods:**

QI results for patients with CC and OC treated in GCCs between 2015 and 2019 are analysed. The median, overall proportion and standard deviation of each QI were calculated. Two-sided Cochran-Armitage tests were applied.

**Results:**

QIs are divided into two categories: process-organization (PO-QIs) and treatment-procedures (TP-QIs), to allow a differentiated analysis for identifying improvement measures.

PO-QIs that reflect the implementation of processes and structures show a high degree of application. PO-QIs have a tremendous influence on the quality of care and are easy to implement through SOPs.

TP-QIs report on treatments that are performed in the GCC. TP-QIs that report on systemic therapies reach a plateau where the guideline is known, but patient-related-factors meaningfully prevent further increase. TP-QIs that report on surgical interventions fluctuate. The most relevant factors are practitioners’ personal skills. Besides the discussion of results amongst peers during the audit, improvement measures could include surgical courses or coaching.

**Conclusion:**

The analysis shows that a combination of different measures is necessary to anchor quality sustainably in health care and thus improve it.

## Introduction

For quality assurance and to implement evidence-based guideline recommendations effectively in everyday oncological care, a ‘Quality Cycle Oncology’ has been established in Germany. Its central elements are defined quality indicators (QIs) derived from strong recommendations of S3 oncological medical guidelines developed by the German Guideline Program in Oncology (GGPO) (Langer and Follmann 2015). The German S3 guidelines are based on a systematic literature review, the presence of a representative interdisciplinary and interprofessional expert panel, including patient advocacy groups, and the use of a formal consensus-building process (Langer and Follmann 2015; Nothacker et al. 2014). An obligatory part of every S3 guideline development process is the definition of QIs from strong recommendations. These are considered suitable as a quality standard since it can be assumed that most patients will gain a clear benefit from the addressed actions of these recommendations. In a multi-step process, interdisciplinary experts of the guideline group identify those strong recommendations of the S3 guideline whose comprehensive implementation improves the provision of care in a defined population and whose ‘translation’ to an indicator is possible (Langer et al. 2017).

The implementation rate of these QIs, and thus the adherence to guideline recommendations, is monitored and evaluated through the certification system implemented by the German Cancer Society (DKG), which serves as one of the core elements of the quality assurance and improvement process for certified cancer centres (Langer et al. 2017).

The results of the QIs are regularly fed back to the GGPO guideline groups to ensure the best possible exchange between the development of evidence- and consensus-based recommendations and clinical routine practice (Beckmann et al. 2016). In the context of guideline updates, the existing quality indicators are also subject to the updating process. Here, the results of the quality indicators are reviewed, and a decision is made as to whether the quality indicator must be retained or changed or, in the case of complete implementation, can be discontinued (Langer et al. 2017).

As of January 2022, 31 tumour-specific and cross-sectional S3 guidelines had been published and 192 quality indicators derived. Thereof, 108 quality indicators are implemented in 18 tumour-specific certification procedures in a total of 1,715 certified centres, including 142 outside of Germany.

In the present study, which was conducted within the scope of a qualifying thesis for a doctorate in medical science at the Charité University Medicine, we present an example from the gynaecological cancer centre (GCC) certification system of the German Cancer Society (DKG).

The certification system for GCCs was developed in 2008 by the DKG and the Working Group for Gynaecological Oncology (Arbeitsgemeinschaft Gynäkologische Onkologie [AGO]) and the German Society for Gynaecology and Obstetrics (DGGG) (Leitlinienprogramm Onkologie. Deutsche Krebsgesellschaft, Deutsche Krebshilfe, AWMF): S3-Leitlinie Diagnostik, Therapie und Nachsorge maligner Ovarialtumoren 2021). As of 2019, a total of 164 GCCs had been certified (Krebsgesellschaft e.V. Jahresbericht der zertifizierten Gynäkolgoischen Krebszentren 2020), and about 55% of all patients in Germany with a first diagnosis (primary case) of a gynaecological tumour^1^ in 2019 were treated in these certified GCC^2^ (Krebsgesellschaft e.V. Jahresbericht der zertifizierten Gynäkolgoischen Krebszentren 2020). Many certified GCC have also joined together in the AGO's working group AG Ovar and are part of the AGO's quality assurance program (QS-OVAR).

Gynaecological tumours consist of several entities that differ in incidence, therapy and prognosis. In 2017, approximately 38,000 women in Germany were diagnosed with a gynaecological neoplasm (Robert Koch Institut 2016).

The GCCs, like all other cancer centres of the DKG, are multidisciplinary and interprofessional networks of qualified partners that represent the entire chain of health care. They commit themselves to adhering to the defined quality standards (i.e., minimum case numbers, tumour boards, high expertise of all network partners, etc.) and transparently disclose the results of their key performance indicators and guideline-derived quality indicators to demonstrate their quality of care and guideline adherence and discuss, if necessary, improvement measures (Mensah et al. 2017).

Especially for gynaecological tumours, various studies have shown that the interdisciplinary cooperation and highly specialised surgical expertise of the clinic and surgeons as well as the surgical case volume have been of great benefit to patients and have had a relevant influence on the clinical outcome (Wright et al. 2011; Bristow et al. 2009; Bois et al. 2009; Munstedt et al. 2003).

The focus of this study will be on two selected gynaecological tumours, namely ovarian and cervical cancers. For both tumour entities, S3 guidelines are available and regularly updated (Leitlinien Programm Onkologie (Deutsche Krebsgesellschaft, Deutsche Krebshilfe, AWMF). S3-Leitlinie Diagnostik, Therapie und Nachsorge maligner Ovarialtumoren; Leitlinien Programm Onkologie (Deutsche Krebsgesellschaft Deutsche Krebhilfe, AWMF). S3-Leitlinie Diagnostik, Therapie und Nachsorge der Patientin mit Zervixkarziom 2021), and in GCCs it has been obligatory to document QIs for these two entities since 2014 for OC and 2015 for CC. For endometrial and vulvar tumours, QIs have been implemented only recently, in 2018 and 2016, respectively, and no S3 guideline is yet available for vulvar carcinoma.

Comprising 3.1% of all malignant neoplasms and 5.2% of all cancer deaths in women, ovarian cancer is the gynaecological cancer with the highest mortality rates (Wesselmann et al. 2014; Robert Koch Institut 2016), representing 19.2% of incident cases of gynaecological neoplasms (Robert Koch Institut 2016). Despite advances in screening and prevention measures, invasive cervical carcinoma, at 11.4% of cases, remains the third most common gynaecological neoplasm in women in Germany and worldwide (Robert Koch Institut 2016; Leitlinien Programm Onkologie (Deutsche Krebsgesellschaft Deutsche Krebshilfe, AWMF). Prävention des Zervixkarzinoms 2020).

Using the example of QIs for ovarian and cervical cancer, this study set out to investigate the development of the implementation rate over time, report results for the time period between 2015 and 2019, evaluate the status of guideline-compliant care and identify areas and corresponding measures to foster improvement. A further goal of this paper is to raise awareness of the potential of guideline-based QIs and their results to contribute to quality assurance and improvement in the clinical routine. The aim is to initiate a discussion and thus jointly define actions and measures to improve health service delivery to ovarian and cervical cancer patients.

## Patients and methods

### Data collection

Each GCC that intends to be (re-)certified must document fulfilment of the requirements. Annually, the results of key performance and quality indicators must be reported to OnkoZert, the independent certification institute that organizes the auditing procedure on behalf of the DKG. After collection from the centres, the datasets are analysed and tested for plausibility. Indicators mostly have target values or defined plausibility limits in which the certified centres have to give a mandatory statement of reasons as to why the limits were overstepped, i.e., in the case of deviation from the guideline recommendation. When target values or plausibility thresholds are reached, centres do not have to give explanations for patients not treated accordingly. For successful certification, cancer centres have to meet the target value or give a plausible explanation if they are not meeting the value (Adam et al. 2018).

Centres are audited regularly by trained gynaecological oncologic medical experts who check the reported data from the previous calendar year before the audit and have insight into patient files during the audit to verify the data. Only verified data are published in the benchmarking reports. For example, 2019 data are audited during 2020 and published in 2021. The data presented here are based on the 2015–2019 patient cohort. Only data from centres that were certified throughout the complete year and had no change in the tumour documentation system are included.

The QIs included in this study are derived according to a defined methodology (German Guideline Program in Oncology (German Cancer Society, German Cancer Aid, Association of the Scientific Medical Societies). Development of guideline-based quality indicators: methodology for the German Guideline Program in Oncology 2021) from the two evidence-based guidelines on the diagnosis, therapy and follow-up of malignant ovarian tumours and patients with cervical cancer published by the GGPO (Leitlinien Programm Onkologie (Deutsche Krebsgesellschaft, Deutsche Krebshilfe, AWMF). S3-Leitlinie Diagnostik, Therapie und Nachsorge maligner Ovarialtumoren 2021; Leitlininien Programm Onkologie (Deutsche Krebsgesellschaft Deutsche Krebhilfe, AWMF). S3-Leitlinie Diagnostik, Therapie und Nachsorge der Patientin mit Zervixkarziom 2021). The treatment guidelines, the corresponding QI and the QI set collected via the certification programme are regularly updated. In this analysis, only QIs that were included in the DKG dataset from 2014 onward and still included as of 2021 were taken into consideration. QIs that had been discontinued over time were not included in this analysis. An overview of discontinued QIs can be seen in (Table 1).

**Table 1:** Discontinued QIs for Ovarian and Cervical Cancer

Indicator	Implementation period	Reason for discontinuation
Ovarian Cancer QIs		
Non-adjuvant chemotherapy of early ovarian carcinoma	2014–2018	Indicator was discontinued due to complete implementation
Platinum-containing chemo-therapy for early ovarian carcinoma	2013–2018	Indicator was discontinued due to complete implementation
Chemotherapy of platinum-resistant and/or refractory first recurrence	2013–2015	Indicator was suspended in the course of the 2015/2016 S3 guideline update due to new recommendations
Combined treatment of platinum-sensitive recurrence	2013–2015	Indicator was suspended in the course of the 2015/2016 update due to new recommendations
No adjuvant therapy BOT (Borderline Ovarian Tumour)	2013–2018	Indicator was discontinued due to complete implementation
Genetic testing offer	2019	Was only included on the data sheet since 2019
Cervical Cancer QIs		
Cisplatinum-containing radio-chemotherapy	2014–2015	Indicator was discontinued due to decision to only include five QIs per tumour entity on the data sheet for certification
Adjuvant radio(-chemo) therapy	2014–2015	Indicator was discontinued due to decision to only include five QIs per tumour entity on the data sheet for certification
Histological confirmation	2014–2015	Indicator was discontinued due to decision to only include five QIs per tumour entity on the data sheet for certification
Spread diagnosis for local recurrence	2014–2015	Indicator was discontinued due to decision to only include five QIs per tumour entity on the data sheet for certification
Pelvic exenteration	2014–2018	Indicator was discontinued due to complete implementation on the data sheet for certification
Complete diagnostic report cervical conization	2021	Will be included in next update of data sheet (Kurzprotokoll zur Sitzung der Zertifizierungskommission Gynäkologische Krebszentren 2017)

https://www.krebsgesellschaft.de/zertkomm-protokolle.html?file=files/dkg/deutsche-krebsgesellschaft/content/pdf/Zertifizierung/Protokolle_Zertkomm/Protokoll%20ZertKomm%20Gyn%207.%20Juni%202016.pdf&cid=32660

### Data analyses

Descriptive analysis of the case distribution, patient numbers and indicator definitions were performed. QI results for patients with cervical cancer (CC) and ovarian cancer (OC) treated in GCCs between 2015 and 2019 were analysed. Only patients from GCCs that had certified status over the entire time period were considered. The median proportion of the centres and overall proportion was calculated for every QI. Two-sided Cochran-Armitage tests were applied to detect trends over time. The standard deviations on the centre level over time were calculated to analyse fluctuations.

Statistical analyses were performed using R version 3.5.1 and the Data-WhiteBox, a data analysis tool developed by OnkoZert. Cochran–Armitage tests were calculated using XLSTAT Version 2019.2.1, excluding centres that had missing values at any reporting point. A *p*-value ≤ 0.05 was considered statistically significant.

The data analysis and study concept were reviewed and approved by the ethics committee of Charité University Medicine in November 2021.

## Results

The number of certified GCCs increased steadily from 2015 to 2019 from 112 to 149, and the number of patients with a primary diagnosis of a gynaecological malignancy treated in GCCs increased from 11,587 to 14,986. Therefore, even though the incidence of OC and CC in Germany has been decreasing over time from 7318 to 7292 and 4606 to 4341, respectively (Robert Koch Institut 2016), the number of patients treated for these two tumour entities has increased in GCCs (OC: 3301–3798 and CC: 2059–2479) (Krebsgesellschaft and e.V. Jahresbericht der zertifizierten Gynäkolgoischen Krebszentren 2020).

The indicators are defined and categorized in (Table 2) including the numerator, denominator and plausibility corridor for the reported QI results. QIs were divided into two categories, (1) process organization (PO-QIs) and (2) treatment procedures (TP-QIs), to allow a differentiated analysis in order to identify areas and corresponding measures to foster improvement in the implementation rate.

**Table 2:** Definition of indicators ovarian carcinoma (numerator, denominator, evaluation of results and category)

Name		Numerator	Denominator	Evaluation of results	Category
Quality indicators for the treatment of ovarian carcinoma					
1	Surgical staging of early ovarian carcinoma	Primary cases of the denominator with surgical staging with –Laparotomy –Peritoneal cytology –Peritoneal biopsies –Bilateral adnexal extirpation –Hysterectomy; where appropriate, extraperitoneal procedure –Omentectomy at least infracolic –Bilateral pelvic and para-aortal lymphonodectomy	Surgical primary cases ovarian carcinoma FIGO I – IIIA	Plausibility corridor > 20%	Treatment procedures
2	Macroscopic complete resection of advanced ovarian carcinoma	Surgical primary cases of ovarian carcinoma FIGO llB-IV with macroscopic complete resection	Surgical primary cases with ovarian carcinoma FIGO IIB-IV	Plausibility corridor > 30% and < 90%	Treatment procedures
3	Surgery of advanced ovarian carcinoma by a gynaecological oncologist	Surgical primary cases of ovarian carcinoma FIGO llB-IV, whose definitive surgical therapy was performed by a gynaeco-oncologist	Surgical primary cases of ovarian carcinoma FIGO llB-IV after conclusion of surgical therapy	Plausibility corridor > 50%	Process organization
4	Post-operative chemotherapy in advanced ovarian carcinoma	Surgical primary cases of ovarian carcinoma FIGO llB-IV with post-operative chemotherapy	Surgical primary cases of ovarian carcinoma FIGO llB-IV and chemotherapy	Plausibility corridor > 30%	Treatment procedures
5	First-line chemotherapy for advanced ovarian carcinoma	Primary cases of ovarian carcinoma FIGO llB-IV with six cycles of first-line chemotherapy carboplatin AUC 5 and paclitaxel 175 mg/m.^2^	Primary cases of ovarian carcinoma FIGO llB-IV	Plausibility corridor > 20%	Treatment procedures

Name		Numerator	Denominator	Evaluation of results	Category
Quality indicators for the treatment of cervical carcinoma					
6	Presentation at the tumour board	Patients (primary cases and ‘non-primary cases’) presented at the tumour board	Patients with an initial diagnosis, recurrence or new remote metastasis of a cervical carcinoma	Plausibility corridor > 20%	Process organization
7	Details in the pathology report on initial diagnosis and tumour resection	‘Surgical primary cases’ of cervical carcinoma with complete pathology reports with details of Histological type according to WHO Grading Detection/non-detection lymph and vein infiltration (L and V status) Detection/non-detection perineural infiltrates (Pn status) Staging (pTNM and FIGO) in the case of conisated patients, bearing in mind the conisation results Depth of invasion and spread in mm in the case of pT1a1 and pT1a2 Three-dimensional tumour size in centimetres (from pT1b1) Minimum distance to the resection margins	‘Surgical primary cases’ with cervical carcinoma and tumour resection	Plausibility corridor > 0.01%	Process organization
8	Details in the pathology report for lymphonodectomy	‘Surgical cases’ with a pathology report containing details of The number of affected lymph nodes in relation to removed lymph nodes Assignment to sampling localisation (pelvic/para-aortal) Details of the widest spread of the largest lymph node metastasis in millimetres/centimetres Details of the detection/non-detection of capsule penetration by lymph node metastasis	‘Surgical cases’ with cervical carcinoma and lymphonodectomy	Plausibility corridor > 0.01%	Process organization
9	Cytological/histological lymph node staging	‘Total cases’ with cytological/histological lymph node staging	‘Total cases’ with cervical carcinoma FIGO stages ≥ IA2–IVA	Plausibility corridor > 0.01%	Treatment procedures

Process organization QIs are defined as indicators that document the implementation of processes and structures explicitly recommended by the medical guideline within the certified network.

Treatment procedure QIs are defined as indicators that report on treatments performed by the members of the certified network, e.g., surgical interventions or recommendations for systemic therapies.

Five QIs were included in the category treatment procedures (four for OC, one for CC) and four QIs in process organization (one for OC, three for CC).

Table 3 presents the results of 9 QIs (5 OC, 4 CC) from 75 GCCs treating 17,495 OC primary cases (incident cases) and 10,969 CC primary cases between 2015 and 2019.

**Table 3:** Quality indicators for ovarian and cervical cancers; treatment years 2014–2019

Indicator			2019 median, absolute patient Nr overall proportion	2018 median, absolute patient Nr overall proportion	2017 median, absolute patient Nr overall proportion	2016 median, absolute patient Nr overall proportion	2015 median, absolute patient Nr overall proportion	2014 median, absolute patient Nr overall proportion	C-A test
Ovarian Carcinoma									
1		Surgical staging of early ovarian carcinoma	81.8% 504/630 80.0%	85.7% 506/647 78.2%	80.0% 485/617 78.6%	85.7% 501/636 78.8%	83.3% 473/603 78.4%	75.0% 384/589 65.2%	0.067
2		Macroscopic complete resection of advanced ovarian carcinoma	75.0% 920/1269 72.5%	68.3% 880/1275 69.0%	69.6% 873/1231 70.9%	70.0% 921/1318 69.9%	62.5% 849/1345 63.1%	58.8% 858/1406 59.9%	0.002
3		Surgery for advanced ovarian carcinoma by a gynaecological oncologist	100.0% 1191/1269 93.9%	100.0% 1192/1275 93.5%	100.0% 1089/1231 88.5%	100.0% 1211/1318 91.2%	92.3% 1166/1345 86.7%	100.0% 1215/1406 86.4%	0.077
4s		Post-operative chemotherapy for advanced ovarian carcinoma	88.9% 923/1130 81.7%	90.9% 914/1117 81.8%	90.0% 954/1081 88.3%	91.7% 1031/1169 88.2%	90.9% 1064/1191 89.3%	94.6% 1157/1265 91.5%	0.021
5		First-line chemotherapy for advanced ovarian carcinoma	60.3% 957/1661 57.6%	61.1% 968/1633 59.3%	63.6% 1004/1559 64.4%	60.0% 1014/1649 61.5%	62.5% 1088/1669 65.2%	69.2% 1113/1649 67.5%	0.022
Cervical Carcinoma									
6	Presentation at the tumour board		100.0% 1857/1913 97.1%	100.0% 1716/1777 96.6%	100.0% 1779/1865 95.4%	100.0% 1695/1777 95.4%	100.0% 1710/1793 95.4%	n/a	0.670
7	Details in the pathology report on initial diagnosis and tumour resection		92.3% 798/874 91.3%	78.4% 652/832 78.4%	68.8% 612/879 69.6%	75.3% 631/890 70.9%	71.3% 648/889 72.9%	n/a	0.001
8	Details in the pathology report for lymphono-dectomy		97.8% 652/669 97.5%	95.0% 667/705 94.6%	90.9% 683/743 91.9%	89.6% 661/735 89.9%	88.0% 706/794 88.9%	n/a	0.170
9	Cytological/histological lymph node staging		72.9% 777/1028 75.6%	78.2% 792/979 80.9%	71.8% 774/1042 74.3%	69.4% 819/1169 70.1%	63.2% 718/1140 63.0%	n/a	0.009

The median is based on the rate of the individual certified centre. The absolute number as well as the overall proportions are based on the cumulative data of all certified centres

C-A Test, Cochran-Armitage test for trend, *p*-value is reported

The implementation rate for PO-QIs that reflect the application of processes and structures either remained stable on a very high implementation level or increased steadily over time to a very high implementation level (e.g., CC: details in pathology report for lymphonodectomy—median 2015: 88.0% to 2019: 97.8%; OC: operation of advanced ovarian carcinoma by a gynaecological oncologist—median 2014: 100.0% to 2019 100.0%).

The implementation rate for TP-QIs that report on treatment methods show an overall high implementation rate, yet the median fluctuates slightly over time (e.g., OC: macroscopic complete resection advanced OC—median 2014: 58.8%; 2015: 62.5%; 2016: 70.0%; 2017: 69.6%; 2018: 68.3.0%; 2019: 75.0%).

Breaking down the TP-QI category further, TP-QIs that address recommendations for systemic therapy show a good to very good implementation rate; however, the analysis indicates that the median is not only fluctuating but decreasing over time (OC: post-operative chemotherapy advanced ovarian carcinoma—median 2014: 94.6% to 2019: 88.9%; OC: first-line chemotherapy of advanced ovarian carcinoma—median 2014: 69.2% to 2019: 60.1%).

By contrast, the overall median for TP-QI results referring to surgical interventions show a good to very good implementation rate, which increased over the past 4 years. The median fluctuates over time (QI 1 surgical staging in early OC—median 2014: 75.0% to 2019: 81.8%; QI 2 macroscopic complete resection advanced OC—mean 2014: 58.8% to 2019: 75.0%).

**Fig. 1:**
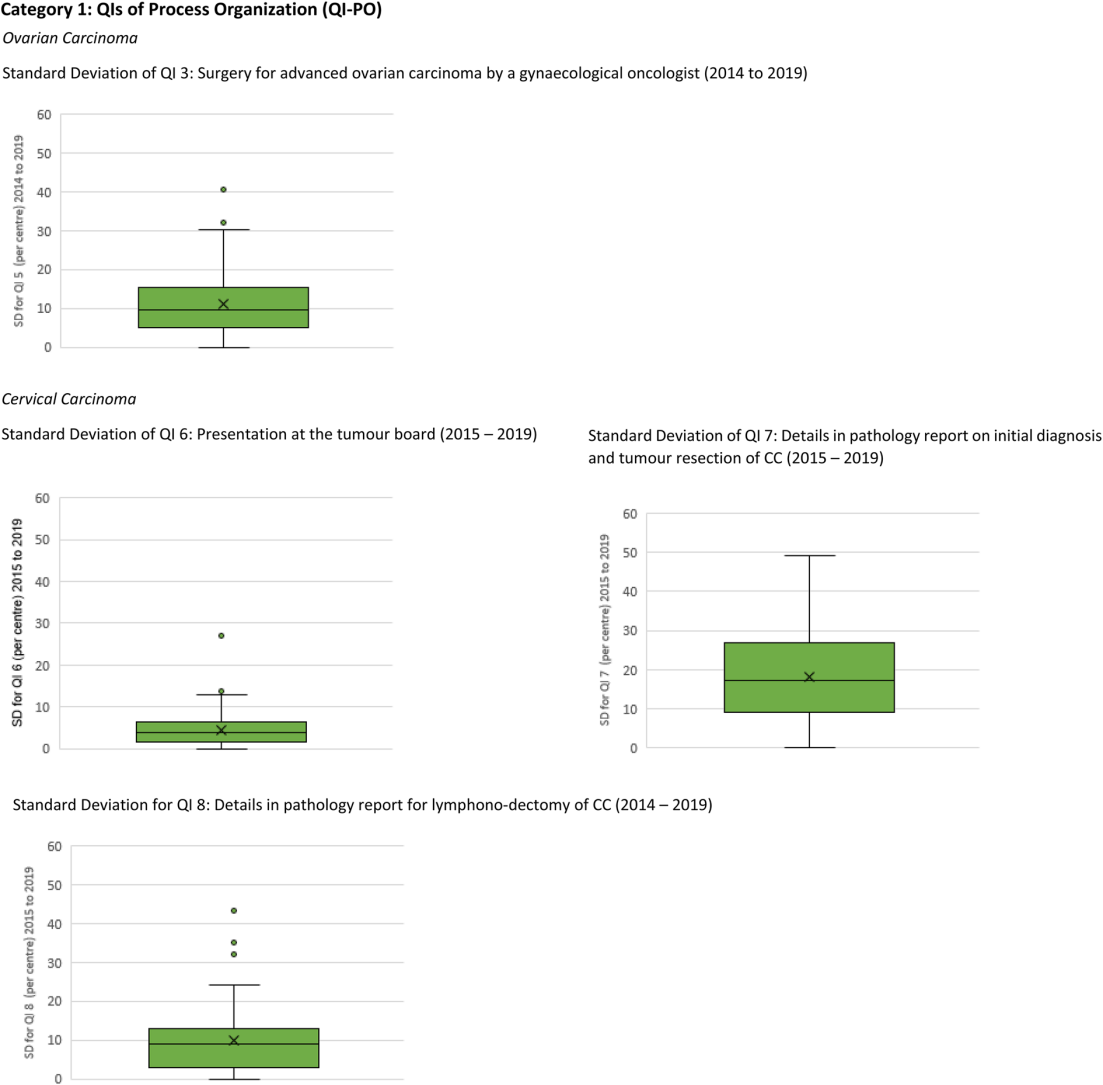

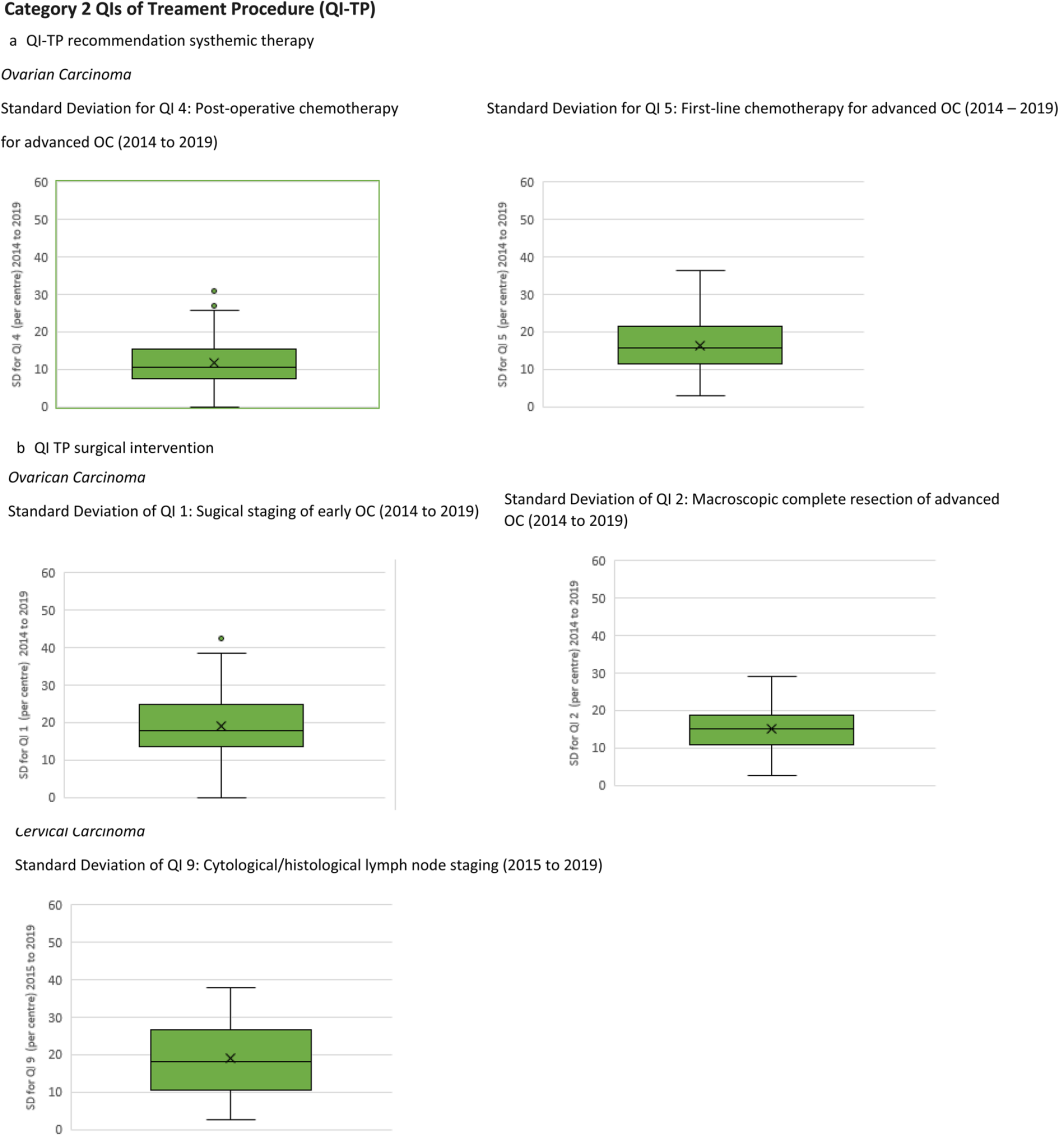
Means of overall standard deviations of centres annual quotas for QIs evaluated between 2014 and 2019

Calculating the SD using the annual QI quota of each centre, the overall mean SD of all QI was calculated and is displayed in a boxplot diagram in (Fig. 1a, b). Analysis of the implementation rate on the individual centre level shows that the results within one centre can vary over time. The mean SD for PO-QIs is the lowest, between 4.4 and 18.2 (e.g., QI 14 presentation at the tumour board CC, mean SD 4.4), the mean SD for TP-QIs that address systemic therapies lies between 11.8 and 16.2 (e.g., QI 12 post-operative chemotherapy for advanced OC, mean SD 11.8), and the mean SD for TP-QIs reporting surgical intervention is the highest, between 15.0 and 19.1 (e.g., QI 1 surgical staging early OC cumulative mean SD 19.1).

The Cochran-Armitage test shows positive trends for five out of nine QI. Positive trends in both categories show four QIs in treatment procedures and one QI in process organization. Trend analyses were conducted over the course of 4 years for the QI 2 ‘macroscopic complete resection advanced OC’, QI 4 ‘postoperative chemotherapy advanced OC’ and QI 5 ‘first-line chemotherapy of advanced OC’. For QI 9 ‘cytological/histological lymph node staging’, the analysis was conducted over the course of 3 years.

## Discussion

This article presents, for the first time, a differentiated overview of the implementation level and development of guideline-derived QI results for OC and CC in certified GCCs.

The results of the evaluated QIs show that the recommendations of the guidelines are implemented to a high or very high extent in the certified GCCs. The quality of care is made visible, and results can be compared between centres. Grouping the analysed QIs into two categories—process organization and treatment procedures—offers the opportunity to assess the improvement potential of QIs in a differentiated way and allows identification of suitable measures for improvement, which can be implemented in the certified centres.

QIs that reflect the implementation of processes and structures within the certified networks are very well applied. The results illustrate that QIs related to procedural aspects have a very high implementation rate (2019: QI 3: 100%; QI 6: 100%, QI 7: 92.3%; QI 8: 97.8%). The excellent implementation rate of this category of QIs has often been realized right from its introduction (e.g., QI 1 and QI 6 each 2015: 100% and 2019: 100%) and is maintained over time. For instance, mandating that surgical therapy for advanced ovarian cancer can only be performed by specialized gynaecologists not only improves outcomes and lengthens survival (Bois et al. 2009; Munstedt et al. 2003; Begg et al. 1998; Junor et al. 1999) but is also easily achievable via a top-down process arrangement. The same process can be applied within the network and to cooperation partners regarding implementation of QI 6 (tumour board presentation rate) and the definition of mandatory information to be included in pathology reports, such as initial diagnosis, tumour resection and, if applicable, indication that lymphadenectomy is complete (QI 7 and QI 8).

These procedural QIs have a tremendous influence on the quality of patient care, while being relatively easy implementable in GCCs, e.g., through standard operating procedures and handling instructions. This is also shown by a consistently high implementation rate and low mean SD of the PO-QI on the individual centre level. Hence, in principle, these indicators and corresponding target values are easily reachable for every certified centre while taking into account justifiable individual cases such as emergency surgery, preventing presentation at the pre-therapeutic tumour board. In the case of repeated not-justifiable non-fulfilment of this indicator group, a ‘deviation’ in the audit will be given. An ultimate failure to fulfil the indicators can lead to withdrawal of the certificate.

Results from QIs that report on treatment procedures such as surgical interventions and recommendations for systemic therapy present a slightly different picture. For evaluation of adherence to recommendations for treatment procedures, it must be considered that situations in routine care are very complex, and conclusions from raw QI data on quality of care are not readily possible (Junor et al. 1999). For example, QI results that do not reach a pre-defined threshold (target value) do not necessarily indicate insufficient performance on the part of the providers. Under such circumstances, additional information is needed to decide whether quality of care is adequate or not (Junor et al. 1999). Therefore, the given explanations by the certified centres are discussed with the auditor during the on-site audit and checked through random samples of patient files. If explanations of the centres seem not to be adequate, the auditors pronounce ‘deviations’ that need to be remedied by the centres (Kowalski et al. 2017). If the explanations are plausible and justifiable, no further action is required.

QIs that call for the implementation of systemic therapies in line with the guideline recommendations show a good yet decreasing implementation rate over time in this analysis (QI 4: 2014 94.6% to 2019 88.9% and QI 5 2014 69.2% to 2019 60.3%). Explanations from the centres that fell below the target value included, for both QIs, mainly patient-related reasons (i.e., patient death after surgery, patient wish, existing comorbidities and/or poor general health, therapy termination due to side effects). For QI 5 (First-line chemotherapy of advanced OC) comorbidities and poor general health often also caused changes in therapy regimes. Patients being treated ex domo / outside the network as well as the time of data reporting (i.e., patients can only be counted in the numerator when the therapy is completed) were named as reasons why patients were missing even though the recommendations for chemotherapy was provided during the tumour boards. It must be kept in mind that written explanations only have to be provided in case the number of patients is below the threshold (QI 4 < 30%; QI 5 < 20%), i.e., if the overall number of eligible patients in the numerator or the median decreases but remains above the threshold, the certified GCCs do not have to provide a reason.

Thus, based on this preliminary evaluation, it can be argued that in contrast to the results of the PO-QIs, the implementation rate for QIs documenting the application of systemic therapies reaches a plateau where the guideline recommendation is known to the practitioners, but patient-related factors prevent a further meaningful increase in the rate. Hence, fluctuations of the implementation rate and higher mean SD of these TP-QIs on the individual centre level are to be expected. The decreasing implementation rate could be in relation to an older age and/or the existence of multiple comorbidities and/or other therapy regimes. Unfortunately, this cannot be further explored with the present data set, as socio-demographic information and detailed information about comorbidities are not yet available or too superficial.

By contrast, TP-QIs that report on surgical interventions offer more room for improvement measures. This set of QIs reflects not only patient-related factors (i.e., comorbidities, poor overall health status, patient rejection of surgery) but also the professional expertise of the surgical team. Surgical therapy is one of the fundamental pillars of the treatment strategy for OC and CC. Not only is it the most important diagnostic instrument; it also has a direct and strong influence on prognosis and is part of a mostly multimodal and interdisciplinary therapy concept (Sehouli et al. 2019). Like QIs reporting on systemic therapy, the data show an increase over time and also reach a plateau in the implementation rate (i.e., QI 1 2014: 75% to 2019 81.8%; QI 2 2014: 58.8% to 2019: 75.0%% and QI 9 2015 63.2% to 2019 72.9%). While keeping in mind that the denominator of the surgical QIs was often small, explanations for not meeting the Q9 (cytological/histological lymph node staging) target value mostly included the application of radio chemotherapy prior to cytological/histological lymph node staging. For QI 2 (macroscopic complete resection of advanced OC), the existence of multiple (distant) metastasis was given as the most frequent reason for an incomplete macroscopic resection. As reported above, some patients also decided to undergo the procedures outside of the certified network. However, besides patient-related topics, the most frequent reasons for not reaching the QI target value included inoperable situs due to advanced spreading of carcinoma or inter-operative assessment, which deemed the surgery as not possible. In the case of QI 2, it was stated several times that the tumour could only be reduced in size but not removed. The data unfortunately do not allow us to assess if other surgical teams would have come to different conclusions and assessments. During the audit, auditors and physicians of the GCC discuss if the results are justifiable, but explanations regarding the deviations are typically brief and often superficial (Inwald et al. 2019).

The following further limitations need to be pointed out in the light of the data interpretation. Firstly, only aggregate data are submitted by the individual centres, hence assessment of individual patients’ information regarding case severity or socio-demographics is not possible. Secondly, the centres included in this analysis could be prone to a selection bias as often only centres that are already performing well join quality assurance programmes. Also, the data investigated here cannot be linked to survival data from registries.

As for these QIs, the most relevant factors are the personal skills of the practitioners, and when these are combined with technical prerequisites, opportunities to identify measures for improvement are given. Thus, measures for improvement of the implementation rate of this QI set, besides the discussion of results amongst peers during the audit, could additionally include offers of surgical courses or coaching.

Interestingly, the data also show that on the individual centre level, the results for macroscopic complete resection, sugical staging of early OC and cytological/hostological LN staging can vary widely from one year to another, with an overall standard deviation of up to 19. Reasons for these fluctuations cannot be provided with the currently available data. When interpreting the results, we must bear in mind the primary purpose of data collection, i.e., creating a basis for the decision of whether or not the certificate should be issued (Inwald et al. 2019). Further investigation is thus necessary. Notwithstanding, one hypothesis could be that, for instance, staff changes in the surgical team could explain why several centres with high indicator results in 1 year can have lower results in the forthcoming year. It could be argued that, meanwhile, the certified GCCs who maintain a constantly high implementation rate provide a good environment for surgeons in training and could be the ones selected to offer coaching courses for other GCCs.

## Conclusion

To achieve the best possible treatment outcomes for women with gynaecological malignancies, synergistic collaboration across all disciplines and professional groups involved in oncological care as well as the pursuit of specialization by physicians are important elements (Wesselmann et al. 2014).

QIs support the establishment of guideline-based treatment in everyday clinical practice and motivate practitioners to critically reflect on their treatment results. In the audit procedures, these results are discussed, and measures are identified that enable better application of the guideline contents. The effectiveness of these measures is reviewed in the next audit 1 year later. The results of the QIs will be reported to the medical guideline development groups and provide information on how and to what extent a recommendation is implemented in everyday clinical practice and thus offer additional suggestions for further development of the guidelines. Furthermore, the results of this analysis, with a focus on ovarian and cervical cancer, suggest that dividing the analysed QI into two categories—process organization and treatment procedures—provides an opportunity to evaluate the QI improvement potential in different ways and allows the determination of appropriate improvement measures and therefore shows that a combination of different measures is necessary to anchor quality sustainably in health care and thus improve it.

## Data Availability

The datasets generated during and/or analysed during the current study are available from the corresponding author on reasonable request.

## Footnotes

1 ICD-10 classifications C48, C51-C57.

2 Results according to ICD-10; Estimated number of new cancer cases in Germany 2017; Centre for Cancer Registry Data at the Robert Koch Institute, www.krebsdaten.de/abfrage, Data status: 30.07.2021. BOT not included because D-diagnosis.
